# Flexible Coordination of Stationary and Mobile Conversations with Gaze: Resource Allocation among Multiple Joint Activities

**DOI:** 10.3389/fpsyg.2016.01582

**Published:** 2016-10-24

**Authors:** Eric Mayor, Adrian Bangerter

**Affiliations:** Institut de Psychologie du Travail et des Organisations, Université de Neuchâtel, NeuchâtelSwitzerland

**Keywords:** multitasking, social interactions, joint action, joint activity, coordination, conversation, communication

## Abstract

Gaze is instrumental in coordinating face-to-face social interactions. But little is known about gaze use when social interactions co-occur with other joint activities. We investigated the case of walking while talking. We assessed how gaze gets allocated among various targets in mobile conversations, whether allocation of gaze to other targets affects conversational coordination, and whether reduced availability of gaze for conversational coordination affects conversational performance and content. In an experimental study, pairs were videotaped in four conditions of mobility (standing still, talking while walking along a straight-line itinerary, talking while walking along a complex itinerary, or walking along a complex itinerary with no conversational task). Gaze to partners was substantially reduced in mobile conversations, but gaze was still used to coordinate conversation via displays of mutual orientation, and conversational performance and content was not different between stationary and mobile conditions. Results expand the phenomena of multitasking to joint activities.

## Introduction

Everyday conversation is perhaps the commonest form of human joint action (Sacks et al., 1974). But its apparent banality belies the intricate processes by which it gets coordinated. Beyond simply producing words for others to hear, conversational participants work together to ensure they understand each other well-enough for current purposes (Clark, 1996). Speakers design utterances tailored to addressees’ knowledge (Sacks et al., 1974) and monitor their signals of understanding (Clark and Krych, 2004), while addressees display their construals of speakers’ utterances (Clark and Schaefer, 1989), e.g., by producing various listener responses like *uh-huh* or *mhm* (Bavelas et al., 2000). Conversational coordination is fundamentally multimodal (Sidnell and Stivers, 2005; Louwerse et al., 2012), relying on both linguistic and embodied signals like gaze, gesture, posture and the like, many of which are interpersonally and cross-modally coordinated (Richardson et al., 2007; Shockley et al., 2009). In particular, gaze plays an important role in coordinating face-to-face conversations (Kendon, 1967; Hanna and Brennan, 2007; Rossano, 2012a). Participants spend much conversational time gazing at each other, with addressees gazing at speakers more often than the reverse (in one study, 75 and 40% of the time respectively; Argyle, 1975). Speakers use gaze to monitor addressees’ understanding (Kendon, 1967) or to elicit a response from them (Bavelas et al., 2002; Rossano, 2012b).

Not only is conversation a highly coordinated joint activity, it also serves to coordinate other joint activities (Bangerter and Clark, 2003) or may co-occur with unrelated joint activities (e.g., talking together while walking together; Mondada, 2014). In everyday conversations, then, perceptual, cognitive and motor resources used for conversation may have to be shared among multiple activities. How does resource sharing happen and what consequences does it entail? Does reduced availability of a resource detract from coordination and impair conversational performance? Or are participants able to flexibly compensate to maintain coordination? We explored these issues for the case of gaze. We chose gaze because prior research suggests it is frequently used in face-to-face conversation and is purportedly normative (Argyle, 1975; Goodwin, 1981); i.e., partners in interaction are expected to gaze at each other often. At the same time, however, cross-cultural data relativizes the role of gaze in social interaction, suggesting that it may not always be normative to gaze at conversational partners (Rossano, 2012b) or that other signals may be used to perform similar functions as gaze (Akhtar and Gernsbacher, 2008). Recent research also suggests flexible use of gaze cues by partners depending on its usefulness (Macdonald and Tatler, 2013). Moreover, engagement in a co-occurring activity like eating may legitimize gaze away from, rather than toward, one’s conversational partner (Wu et al., 2013). Rossano (2012b) showed that a participant engaged in cooking pasta on a stove while listening to a story periodically shifted his gaze back and forth between the narrator and the stove, thus displaying an orientation to the narrator’s talk while cooking. These data raise the possibility that while gaze patterns in multiple joint activities may differ from those in a single joint activity, gaze may continue to serve an important coordination function, namely displaying a mutual orientation to the conversation.

Multiple concurrent joint activities are frequent in everyday life but currently poorly understood. Theories of multitasking (e.g., Salvucci and Taatgen, 2008) describe resource allocation processes between multiple task threads in detail, including how conflicts among resources may occur, leading to interruptions (McFarlane and Latorella, 2002) or task switching (Monsell, 2003). But such research remains focused on individual cognition and action. In joint activities, however, the same resources (like gaze in the present case) may be required for both cognitive and motor control of individual actions and communicative coordination between participants, including displays of mutual orientation to the joint activity. To be complete, theories of multitasking thus need to incorporate both individual and joint processes, i.e., communication processes as well as cognition (Haddington et al., 2014). A consideration of multitasking as joint action also expands the scope of coordination processes beyond issues of control to encompass managing the identities and affiliations (Enfield, 2006) of interactional partners. This kind of issue is particularly salient in cases where participants in multiple joint activities have to suspend one activity to attend to another, emergent solicitation (Chevalley and Bangerter, 2010). For example, when Ann suspends a conversation with Björn in order to deal with an incoming phone call from Camilla, it is necessary to manage the face needs (Brown and Levinson, 1987) of the partners (e.g., Ann might apologize for the interruption and possibly justify it to Björn). This is because engaging in joint activities requires participants to commit their resources to furthering the joint activity, and thus they are accountable to each other for the proper use of those resources (Chevalley and Bangerter, 2010).

There is little data that speak to these issues. Field studies exist, primarily from the tradition of conversation analysis. In an edited volume (Haddington et al., 2014), various aspects of the coordination of multiple activities were investigated, focusing on the interplay between verbal and embodied resources in the real-time unfolding of those activities. A variety of settings have been studied, including talking while driving (Mondada, 2012) and nursing teams (e.g., Mayor and Bangerter, 2015). Field studies can describe how resource sharing among activities is accomplished, which is a valuable contribution. But for a complete understanding of how multiple joint activities are coordinated, experimental data is also needed (Clark and Bangerter, 2004; Brennan, 2005). For example, only experimental studies can investigate the consequences of resource allocation to different concurrent tasks, e.g., potential detrimental effects on coordination. However, by their very nature, multiple joint activities are difficult to study experimentally. Bangerter et al. (2010) and Chevalley and Bangerter (2010) combined corpus and experimental data to build and test a model of how participants suspend a primary joint activity when one participant needs to allocate all of his or her resources to another one, and how they subsequently reinstate the primary activity.

In the current study, we created a novel experimental paradigm to investigate the case of talking while walking, a common conjunction of joint activities in everyday life. Walking together requires coordinating speed, posture, and gait. Synchronizing gait is accomplished via tactile (hand-holding) or visual signals (Zivotofsky and Hausdorff, 2007). When people walk somewhere together, they may also turn, which also requires coordination. All of these processes involve processing visual information via gaze. Thus, in walking while talking, gaze is a resource that gets shared between conversation and the demands of joint mobility. We investigated three research questions. How is gaze allocated among constraints arising from these concurrent activities? Does allocation of gaze to other, mobility-related activities affect conversational coordination in terms of displays of mutual orientation? Does reduced availability of gaze for conversational coordination affect conversational performance?

In our experiment, pairs of participants conversed in four within-subjects conditions of varying mobility. In the *talk-only* condition, one participant, the narrator, told a story to the other participant, the listener, while both were standing immobile. In the *talk-and-walk* condition, the narrator told another story to the listener while they walked together along a straight-line itinerary. In the *talk-and-navigate* condition, the narrator told another story to the listener while they walked together along an itinerary that featured five changes of direction. In the *navigate-only* condition, participants walked together along an itinerary that featured five changes of direction, but without having to perform the storytelling task (we did not add a control condition corresponding to the talk-and-walk condition (i.e., a walk-only condition) to maintain the feasibility of the study at a manageable level). In the three mobile conditions, either the narrator or the listener was designated as the *navigator* (between-subjects condition) and was entrusted with a map of the itinerary. The experiment thus implemented a 4 (mobility; within-subjects) × 2 (navigator; between-subjects) factorial design.

First, we wanted to measure the decrease in gaze to one’s partner in mobile conditions relative to the baseline constituted by the stationary condition. Currently, there is no data available on how often people gaze at each other in mobile conversations. Moreover, it is also unclear whether the asymmetry in gaze to the partner between speakers and listeners in stationary conversation (Argyle, 1975) also holds in mobile conversation. In each mobility condition, we thus investigated gaze allocation by the narrator and the listener to three targets: (1) the partner, (2) the map, and (3) elsewhere (e.g., the path). Gaze to the partner in the talk-only condition serves conversational coordination. In the three mobile conditions, it may serve conversational coordination or other purposes (e.g., coordinating navigation). Therefore, the proportion of gaze to the partner in the three mobile conditions is an upper-bound estimate of the use of gaze to coordinate conversation while walking together.

Second, we assessed whether gaze to the partner in mobile conversations still serves conversational coordination, focusing on the talk-and-walk and the talk-and-navigate conditions because they instantiate clear multitasking situations. In other words, even if gaze to partners is reduced in mobile conversations, can similar patterns be demonstrated or does gaze occur randomly? Because conversational participants display an ongoing mutual orientation to the conversational activity (Goodwin and Heritage, 1990), both speakers and listeners collaborate to achieve that mutual orientation and gaze is a primary means to do so. This principle is applied differently by speakers and listeners, however (Ho et al., 2015). On the one hand, speakers use gaze initiation toward listeners to pursue a response from them (Bavelas et al., 2002; Rossano, 2012b). In our study, it follows that, if there is mutual orientation, narrator gaze initiation to listeners should increase the likelihood of a response from the listener. Because face-to-face conversation is multimodal, this response can be produced either verbally (a back-channel like *uh-huh* or a similar acknowledging utterance), or visually (gaze initiation by the listener to the narrator). We call this the listener-response hypothesis. On the other hand, listeners engaged in monitoring another activity concurrent to conversation shift gaze back and forth between speakers (to display recipiency) and that other activity (Rossano, 2012b). In our study, it follows that listeners will periodically look at narrators. Therefore, they are less likely to gaze at narrators if they have recently gazed at them. We call this the periodic-monitoring hypothesis. Evidence for these two hypotheses would suggest that even reduced gaze to the partner in mobile conversations is not random but serves conversational coordination via displays of mutual orientation to the ongoing conversation.

Third, we assessed conversational performance and content in mobile and stationary conversations. If performance and content remain comparable in spite of reduced availability of gaze, this would constitute evidence for the ability of conversational partners to flexibly accommodate conversation to joint multitasking situations. We assessed performance by measuring behaviors typical to the narrator and listener roles. Narrators’ main role in the conversational task is telling stories, and thus an important performance indicator is speech fluency. Narrator disfluencies are thus a negative measure of the quality of delivery of a narrative. Listeners’ main role in the conversational task is to display active participation, and the frequency of listener responses is thus an important performance indicator. Note that both indicators may be correlated; distracted listeners produce less responses, which in turn affects the quality of the narrative produced (Bavelas et al., 2000), and thus these measures are good indicators of whether participants may be distracted by a lack of mutual orientation to the conversation. We thus assessed whether narrator disfluencies and listener responses vary between the stationary and mobile conditions. Finally, we also measured whether stationary and mobile conversations differed in content. As a measure of content, we compared the relative frequency of affect and cognition words in the narrators’ speech across experimental conditions using the LIWC software package (Pennebaker et al., 2001) which enables automated content analysis of texts.

## Materials and Methods

### Ethics Statement

This study has been conducted in accordance with the guidelines of the Swiss Psychological Society.

### Participants

Eighty participants (*n* = 40 pairs, native French speakers, 17 men for each between-subject condition) were recruited on campus or by e-mail and paired with people they did not know. They gave informed consent and received 20 Swiss Francs each for participating. Sample size was determined according to prior practice for experimental conversational studies (Richardson et al., 2007). We ran data collection until the planned *n* was attained. Data from six pairs had to be replaced because of recording problems.

### Procedure

The experiment took place outdoors in a quiet urban environment. Participants conversed together in four different mobility conditions described above. Participants were randomly assigned to the narrator or listener role, and either the narrator or listener was randomly assigned to the navigator role. The order in which pairs performed each mobility condition was counterbalanced.

**FIGURE 1:**
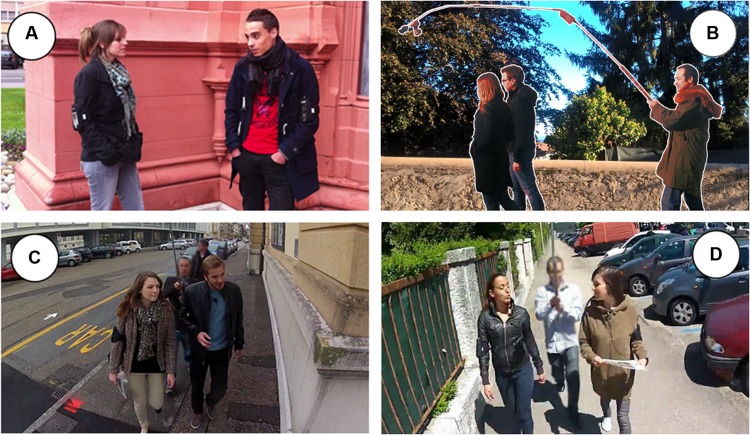
**(A)** Talk-only condition. **(B)** Lateral view of the perch. **(C)** Talk-and-navigate condition, pair turning to their left. **(D)** Talk-and-navigate condition, pair engaged in mutual gaze.

In the talk-only condition, participants’ conversation was videotaped in HD via an iPhone 4 at a distance of approximately 1.5 m (**Figure 1A**). In the remaining (mobile) conditions, we videotaped each pair in HD while they walked using a GoPro Hero2 camera mounted on a perch held by an experimenter walking 1 m behind the pair (**Figure 1B**). This setup afforded a moving frontal view of the participants (**Figures 1C,D**), enabling reliable coding of gaze (e.g., **Figure 1D**). In the three mobile conditions, navigators were given an A4-size map (a Google maps printout) with the itinerary indicated in red. Both participants were instructed to follow the itinerary together. Navigators were further told they were responsible for the itinerary. Each participant was also equipped with an audio recorder and a tie microphone as a backup.

### Data Preparation

**FIGURE 2:**
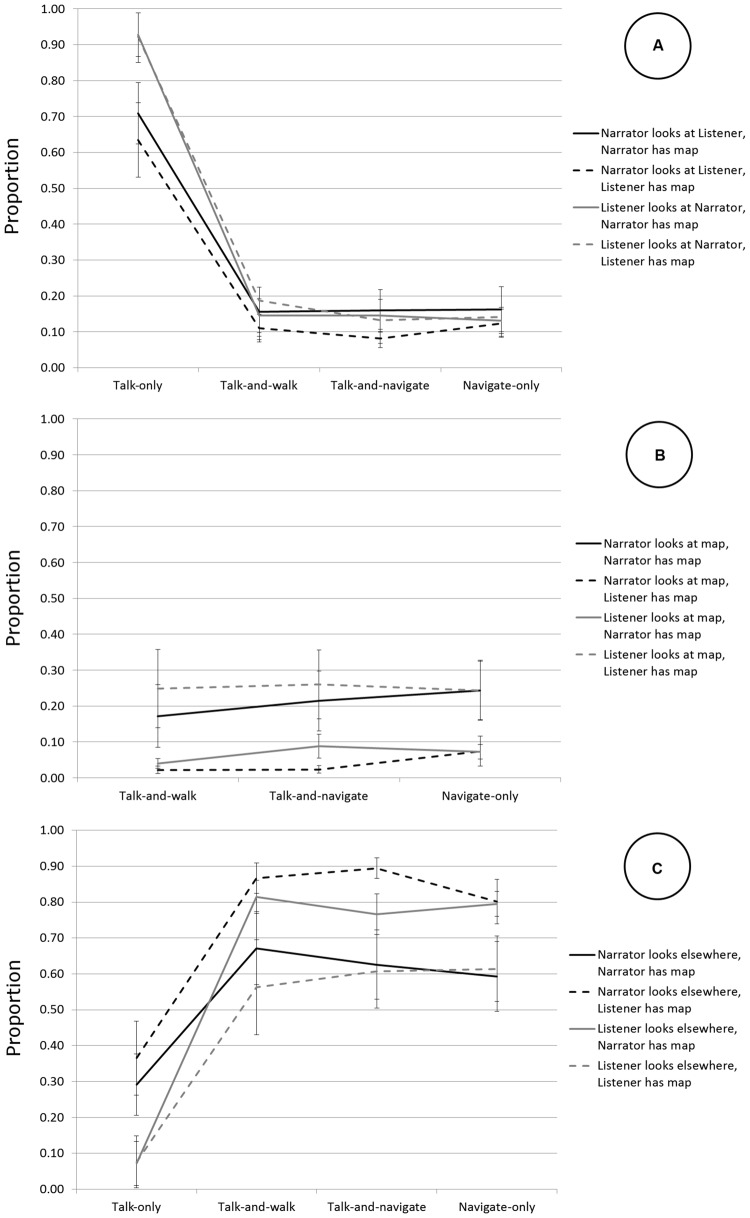
**Gaze allocation in stationary and mobile conversations. (A)** Proportion of gaze at partner. **(B)** Proportion of gaze at map. **(C)** Proportion of gaze elsewhere. Error bars represent the standard error of the mean.

Investigating the listener-response hypothesis and periodic-monitoring hypothesis requires synchronized multimodal time course data on the level of the second. Using snapshots of the video files, each participant’s gaze for each second of the task was coded as either directed to the other person (typically, the participant’s head is turned to the partner, with the lower jaw more or less horizontal), to the map or elsewhere (a 1-s window ensures sufficient granularity because gaze duration to partners in conversation typically is around 3 s, Cook, 1977). Double-coding of gaze direction was performed on 797 snapshots. Interrater agreement was good, Cohen’s κ = 0.754 (*p* < 0.001) for narrator’s gaze and 0.824 for listener’s gaze (*p* < 0.001). We also computed automatically, for each second of video, whether or not the listener had gazed at the narrator within the preceding 5 s (testing the periodic-monitoring hypothesis). We chose a 5-s window because it corresponds to the approximate average proportion of gaze to the partner (**Figure 2A**). We also coded, as control variables, for each second of video, whether a change of direction started or not (κ = 0.91, 3-s window), and whether the end of the itinerary was reached or not.

Conversations were transcribed word-for-word. For each second of video, we coded whether or not a verbal listener response (*okay*, *mhm*, short replies; Bavelas et al., 2002) related to the storytelling activity was initiated (i.e., we did not code potential listener responses related to conversations about navigation). We double-coded 10% of the data (four pairs) to compute interrater agreement, which was good (κ = 0.74). We coded the number of narrator disfluencies, measured as filled pauses (*uh*, *um*; Clark and Fox Tree, 2002) or repetitions (e.g., *th- the*). Interrater agreement, computed on the base of the same four pairs, was good (*r* = 0.80). We then computed the rate of listener responses and narrator disfluencies per 100 words. We used LIWC (Pennebaker et al., 2001) to compute the rate of affect and cognition words per 100 words of narrators’ speech in each condition. Affect words (e.g., *cry*, *sad*, *happy*) index emotional content in speech. Cognition words (e.g., *think*, *consider*, *because*) index reasoning in speech.

## Results

### Gaze Allocation in Stationary and Mobile Conversation

The proportions of time spent gazing to partners, the map and elsewhere are shown in **Figure 2**. We performed a 4 (condition) × 2 (conversational role) × 2 (navigator role) mixed ANOVA on the proportion of time spent gazing at the partner. In the three mobile conditions, we performed a 3 (mobile condition) × 2 (conversational role) × 2 (navigator role) mixed ANOVA on the proportion of time spent gazing at the map. We did not perform analyses on the proportion of time spent gazing elsewhere because this target is theoretically less interesting and the additional ANOVA would be somewhat redundant given the interdependent nature of proportions. We used the Greenhouse–Geisser correction for degrees of freedom when the sphericity assumption was violated.

For gaze to partners, there was an effect of condition, *F*(1.91,72.63) = 514.50, *p* < 0.001 ($\eta_{p}^{2}$ = 0.931), all mobile conditions differed from the stationary condition, all *F*s < 618.57, all *p*s < 0.001 (all $\eta_{p}^{2}$s > 0.942). Participants gazed more at partners in the stationary condition than in any of the mobile conditions. There was no significant effect of navigator role, *F*(1,38) = 0.787, *p* = 0.38 ($\eta_{p}^{2}$ = 0.02), nor was there a significant interaction between condition and navigator role, *F*(1.91,72.63) = 0.51, *p* = 0.594 ($\eta_{p}^{2}$ = 0.01). The interaction between conversational role and condition was significant: *F* (1.52,57.8) = 32.13, *p* < 0.001 ($\eta_{p}^{2}$ = 0.458). Listeners gazed at narrators more often than the reverse in the talk-only condition but not in the mobile conditions, as shown by contrast analyses (all interaction *F*s > 26.05, all *p*s < 0.001, all $\eta_{p}^{2}$s > 0.40).

For gaze at the map, there was a main effect of condition, *F*(2,76) = 5.16, *p* = 0.008 ($\eta_{p}^{2}$ = 0.120). Participants gazed at the map more in the navigate-only condition than in the talk-and-walk-condition, *F*(1,38) = 8.207, *p* = 0.007 ($\eta_{p}^{2}$ = 0.18). There was no effect of conversational role, *F*(1,38) = 1.45, *p* = 0.24 ($\eta_{p}^{2}$ = 0.04), or navigator role, *F*(1,38) = 0.045, *p* = 0.83 ($\eta_{p}^{2}$ = 0.001). The interaction between condition and navigator role was not significant, *F*(2,76) = 1.40, *p* = 0.254 ($\eta_{p}^{2}$ = 0.035). There was an interaction between condition and conversational role, *F*(2,76) = 3.62, *p* = 0.032 ($\eta_{p}^{2}$ = 0.09): narrators gazed more at the map in the navigate-only condition than in the talk-and-walk condition, whereas listeners gazed equally often at the map in both conditions, *F*(1,38) = 4.261, *p* = 0.046 ($\eta_{p}^{2}$ = 0.101). Finally, the interaction between conversational role and navigator role was significant, *F*(1,38) = 39.18, *p* < 0.001 ($\eta_{p}^{2}$ = 0.51). Narrators gazed more at the map when they were navigators than when they were not, and the same was true for listeners.

In summary, both participants gazed at each other significantly less often when mobile than when stationary (**Figure 2A**). When aggregating over the three mobile conditions, mobility reduced gaze to one’s partner from 80% of the time to 14% of the time. Mobile participants gazed elsewhere on average 71% of the time, compared with 21% of the time in the stationary condition (**Figure 2B**). They gazed at the map 23.1% of the time when they were navigators and 5.4% of the time when they were not (**Figure 2C**). While listeners gazed at narrators more than the reverse in the stationary condition (similar to Argyle, 1975), there was no evidence of such an asymmetry in the mobile conditions.

### Gaze and Coordination in Mobile Conversations

To test the listener-response hypothesis and the periodic-monitoring hypothesis, we conducted mixed-model logistic regression analyses in R 3.1. This allowed taking the interdependence of observations within pairs into account. We used logistic regression because the dependent variables are binary. The analysis for each hypothesis was run separately for the talk-and-walk and for the talk-and-navigate conditions because the control variables are different in these conditions. In all analyses, pairs were entered as random effects.

The first analysis tests the listener-response hypothesis for verbal behavior. The dependent variable is whether a verbal listener response occurred or not at each second of conversation. The main predictor was whether or not the narrator had initiated gaze to the listener in the preceding 2 s (we chose a 2-s window because it corresponds approximately to the average duration of narrator gaze to listeners in the mobile conditions). In the talk-and-walk condition, we also entered whether or not the end of the itinerary was imminent (i.e., coming up in 1 s or less) or not as a control variable. Likewise, in the talk-and-navigate condition, we also entered whether or not the end of the itinerary or a change of direction was imminent (i.e., coming up in 1 s or less) as control variables. Results are shown in **Table 1**, where the estimates for each predictor have been converted into odds ratios that show the increase or decrease in likelihood of a listener response when the predictor is present.

**Table 1:** Mixed-effects logistic regression summaries for predictors testing the listener response hypothesis for verbal behavior (recent narrator gaze as predictor for verbal listener response) in two conditions.

		Estimate	95% CI	OR	*p*
Talk-and-walk condition					
	Intercept	-2.62	-2.88 to -2.37	0.07	<0.001
	Listener is navigator	0.37	0.02 to 0.72	1.45	0.04
	Recent narrator gaze	0.35	0.18 to 0.52	1.42	<0.001
	End imminent	-13.30	-22.73 to 3.87	<0.01	0.01
Talk-and-navigate condition					
	Intercept	-2.88	-3.17 to -2.59	0.06	<0.001
	Listener is navigator	0.57	0.19 to 0.98	1.80	<0.001
	Recent narrator gaze	0.31	0.13 to 0.49	1.36	<0.001
	Turn imminent	-0.02	-0.52 to 0.49	0.98	0.95
	End imminent	-17.58	-143.03 to 107.86	<0.01	0.78

*Odds ratios (ORs) are computed as exp(Estimate). ORs significantly lower than 1 mean that the likelihood of gaze initiation decreases when the predictor is present. ORs significantly higher than 1 mean that the likelihood of gaze initiation increases when the predictor is present. This is compared to the intercept which is the odds of listener response initiation when all predictors have a value of 0.*

The main result in **Table 1** is that in both the talk-and-walk and the talk-and-navigate conditions, narrator gaze to listeners significantly increases the likelihood of a listener response within 2 s (odds ratios of 1.42 and 1.36 respectively in each condition), thus supporting the listener-response hypothesis. In the talk-and-walk condition, listener responses were also more likely when the listener was the navigator (odds ratio of 1.45) and were less likely when the end of the itinerary was imminent (odds ratio < 0.01). In the talk-and-navigate condition, listener responses were also more likely when the listener was the navigator (odds ratio of 1.8).

The second analysis tests the listener-response hypothesis for gaze as well as the periodic-monitoring hypothesis. The dependent variable is whether or not listener gaze initiation to the narrator occurred at each second of conversation. The main predictors were whether or not the narrator had initiated gaze to the listener in the preceding 2 s (for the listener-response hypothesis) and whether or not the listener had initiated gaze to the narrator in the preceding 5 s (for the periodic-monitoring hypothesis). As for the previous analyses, we also entered whether the end of the itinerary was imminent or not (talk-and-walk and talk-and-navigate conditions) and whether a change of direction was imminent or not (talk-and-navigate condition) as control variables. Results are shown in **Table 2**.

**Table 2:** Mixed-effects logistic regression summaries for predictors testing the listener response hypothesis for non-verbal behavior (recent narrator gaze as predictor of listener gaze initiation), and the periodic-monitoring hypothesis (recent listener gaze as predictor for listener gaze initiation) in two conditions.

		Estimate	95% CI	OR	*p*
Talk-and-walk condition					
	Intercept	-2.46	-2.84 to -2.07	0.09	<0.001
	Listener is navigator	-0.24	-0.78 to 0.30	0.79	0.38
	Recent narrator gaze	0.21	0.03 to 0.40	1.23	0.03
	Recent listener gaze	-0.82	-0.99 to -0.66	0.44	<0.001
	End imminent	1.19	0.38 to 1.99	3.29	<0.001
Talk-and-navigate condition					
	Intercept	-2.49	-2.94 to -2.03	0.08	<0.001
	Listener is navigator	-0.46	-1.10 to 0.18	0.63	0.16
	Recent narrator gaze	0.34	0.15 to 0.52	1.40	<0.001
	Recent listener gaze	-0.69	-0.84 to -0.54	0.50	<0.001
	Turn imminent	-0.32	-0.94 to 0.29	0.72	0.31
	End imminent	0.45	-0.60 to 1.51	1.58	0.40

*Odds ratios (ORs) are computed as exp(Estimate). ORs significantly lower than 1 mean that the likelihood of listener’s gaze initiation decreases when the predictor is present. Odds ratios significantly higher than 1 mean that the likelihood of listener gaze initiation increases when the predictor is present. This is compared to the intercept which is the odds of gaze initiation when all predictors have a value of 0.*

The listener-response hypothesis for gaze was supported: in both the talk-and-walk and the talk-and-navigate conditions, narrator gaze initiation to listeners significantly increases the likelihood of listener gaze initiation within 2 s (odds ratios of 1.23 and 1.4 respectively in each condition). The periodic-monitoring hypothesis was supported as well: in both the talk-and-walk and the talk-and-navigate conditions, listener gaze initiation was significantly less likely if the listener had initiated gaze to the narrator in the preceding 5 s (odds ratios of 0.44 and 0.50 respectively in each condition). In addition, we also found that an imminent end of the itinerary significantly increased the likelihood of listener gaze to the narrator in the talk-and-walk condition (odds ratio of 3.29).

### Performance and Content of Stationary and Mobile Conversations

Even though residual gaze in mobile conversations serves conversational coordination via displays of mutual orientation, its reduced availability might adversely affect performance of mobile conversations. We investigated whether listeners were less responsive and whether narrators were less fluent in the mobile conditions than in the stationary condition, and also compared content across conditions. We first computed the rate of verbal listener responses produced by the listener per 100 words produced by the narrator. Because the navigate-only condition does not have listener and narrator roles, we performed a 3 × 2 mixed ANOVA to test the effect of the other three conditions and navigator role on the rate of listener responses. We used the Greenhouse–Geisser correction for degrees of freedom when the sphericity assumption was violated. The rate of listener responses was not significantly affected by condition: *F*_Condition_(1.41,53.88) = 1.089, *p* = 0.325 ($\eta_{p}^{2}$ = 0.028). It was affected by navigator role: *F*_Role_(1,38) = 5.606, *p* = 0.023 ($\eta_{p}^{2}$ = 0.129), *M* = 2.911 (*SD* = 1.43, 95% CI: 2.773–3.049) when the narrator was the navigator, compared with *M* = 4.129 (*SD* = 2.10, 95% CI: 3.457–4.801) when the listener was the navigator. Interestingly, then, listeners produced significantly more verbal listener responses when they were navigators than when they were not (see above). It is unclear why they did so, although they may have done so to verbally display increased orientation to the conversation to compensate for their increased gaze to the map (**Figure 2B**).

We then tested whether the disfluency rate varied for the narrator by condition. We performed a 4 × 2 mixed ANOVA to test the effect of condition and navigator role on the rate of disfluencies produced by the narrator. Condition affected disfluencies: *F*_Condition_(3,114) = 18.333, *p* < 0.001 ($\eta_{p}^{2}$ = 0.325). Contrast analyses show that only the difference between the talk-only and the navigate-only condition is significant (*p* < 0.001). The rate of disfluencies was on average 2.832 (*SD* = 1.251, 95% CI: 2.432–3.232) in the talk-only condition, 2.94 (*SD* = 1.044, 95% CI: 2.606–3.274) in the Talk and walk condition, 2.966 (*SD* = 1.116, 95% CI: 2.609–3.323) in the talk-and-navigate condition and finally 1.860 (*SD* = 0.088, 95% CI: 1.832–1.888) in the navigate-only condition. Navigator role did not significantly affect disfluencies: *F*_Role_(1,38) = 0.069, *p* = 0.795 ($\eta_{p}^{2}$ = 0.002).

We then tested whether the rate of affect and cognition words per 100 words varied for the narrator by condition and navigator role (4 × 2 mixed ANOVAs separately for affect and cognition words). There was an effect of condition on the affect words rate: *F*_Condition_(3,114) = 9.514, *p* < 0.001 ($\eta_{p}^{2}$ = 0.200). Contrast analyses show that only the difference between the talk-only and the navigate-only condition is significant (*p* < 0.001). The rate of affect words was on average 4.429 (*SD* = 1.253, 95% CI: 4.028–4.830) in the talk-only condition, 4.64 (*SD* = 1.224, 95% CI: 4.252–5.034) in the talk-and-walk condition, 4.437 (*SD* = 1.189, 95% CI: 4.056–4.818) in the talk-and-navigate condition and finally 5.793 (*SD* = 1.800, 95% CI: 5.217–6.369) in the navigate-only condition. There also was a marginally significant effect of navigator role: *F*_Role_(1,38) = 3.765, *p* = 0.060 ($\eta_{p}^{2}$ = 0.095). There was an effect of condition on the cognition words rate: *F*_Condition_(2.169,114) = 3.973, *p* = 0.020 ($\eta_{p}^{2}$ = 0.095). Contrast analyses show that only the difference between the talk-only and the navigate-only condition is significant (*p* < 0.006). The rate of cognition words was on average 20.368 (*SD* = 2.289, CI = 19.636–21.100) in the talk-only condition, 20.340 (*SD* = 2.528, CI = 19.532–21.148) in the talk-and-walk condition, 20.020 (*SD* = 2.400, CI = 19.244–20.780) in the talk-and-navigate condition and finally 18.839 (*SD* = 3.607, CI = 17.685–19.993) in the navigate-only condition. There was no effect of navigator role: *F*_Role_(1,38) = 0.291, *p* = 0.593 ($\eta_{p}^{2}$ = 0.008).

In summary, there are few differences in the rate of narrator disfluencies, listener responses and the affective and cognitive content of conversations. Most notably, there are no systematic differences between stationary and mobile conditions, suggesting that conversational performance and content was not affected by mobility. The difference in affective and cognitive content between the talk-only and the navigate-only condition may be due to the fact that, in the navigate-only condition, there was no storytelling requirement, so participants engaged in small talk.

## Discussion

In this study, we aimed at contributing to a better understanding of how conversational resources are allocated among multiple joint activities, focusing on the case of gaze. We assessed how gaze gets allocated among various targets in stationary and mobile conversations, whether allocation of gaze to other targets in mobile conversations affects conversational coordination, and whether reduced availability of gaze for conversational coordination in mobile conversations affects conversational performance and content.

Participants’ gaze to their partner decreased substantially when moving from stationary to mobile conversation. This constitutes evidence that the addition of another concurrent joint activity leads to a reallocation of multimodal resources among the two activities. It may also be the case that the decrease in gaze in mobile conversations partly reflects the shoulder-to-shoulder body orientation of the participants, and not the demands of mobility *per se*. Participants who talk together often spontaneously adopt an “L-arrangement” (Kendon, 1990) with their bodies oriented toward each other at a 90° angle (as in **Figure 1A**). Participants in a different arrangement (e.g., sitting side-by-side on a bench) may naturally gaze less at each other, independently of any coordination demands of joint mobility. There is evidence that interactional configurations (F-formations; Kendon, 1990) affect gaze frequency. For example, participants gaze less at each other when their physical proximity increases (Argyle and Dean, 1975). However, participants spontaneously and flexibly adapt their interactional configurations to the activity or activities they are currently performing together. As a result, the decreased gaze in mobile conversations remains an effect (albeit an indirect effect) of the necessity of reallocating gaze among concurrent joint activities.

Despite the reduced availability of gaze, it was still used to coordinate mobile conversations in two ways. First, narrator gaze triggered both verbal and non-verbal responses in listeners. Second, listeners were less likely to gaze at the narrator if they had recently done so, suggesting that they used gaze to periodically monitor the conversation (of course, this finding does not exclude other potential causes). There were no systematic differences in performance and content of stationary and mobile conversations. These findings show that frequent gaze is not always necessary to maintain conversational coordination in face-to-face situations. High rates of gaze in stationary conversations (Argyle, 1975) may reflect situation-specific conversational norms (Rossano, 2012b) more than actual coordination requirements. In particular, in the course of multiple concurrent joint activities, participants may exhibit a shared awareness of the contingencies of those activities, and thus be more tolerant of deviations from habitual forms of coordination employed in single joint activities. Use of multimodal conversational signals may thus be more flexible than previously assumed, with participants being able to allocate them to competing activities while continuing to display an orientation to the main conversational activity and without jeopardizing conversational performance.

Our study successfully used a novel experimental paradigm to explore the two concurrent joint activities of walking and talking. There are some limitations, however. First, while we were able to reliably code gaze to various targets, we were not able to measure the potential awareness of the partner’s actions mediated by peripheral vision. Peripheral vision might have allowed both speakers and listeners to monitor mutual orientation to the conversation without overt gaze to one another. Second, while we measured conversational performance in terms of listener responses and speaker disfluencies, it may well be the case that other aspects potentially related to performance (e.g., listener comprehension of the story) or to the social relationship between the participants (e.g., trust or empathy) may have been detrimentally affected by the reduced availability of resources like gaze in mobile conversations.

Further research might investigate how other multimodal resources (e.g., gesture) are allocated between co-occurring joint activities. Some resources might be used in multimodal tradeoffs to compensate for the decreased availability of another resource. An example of this is our finding that listeners who were navigators produced significantly more verbal listener responses than listeners who weren’t. This might reflect a means of compensating for the decreased availability of gaze as a way of demonstrating orientation to the conversation. Further research might also investigate conditions under which coordination demands of multiple co-occurring joint activities exceed available resources of participants. In our data, there were several cases where demands related to the coordination of emergent walking-related activities required participants to suspend the storytelling task, for example, in order to figure out when to change direction (Mayor and Bangerter, 2013). In such situations, participants suspend one of the joint activities in order to allocate all resources to the other activity before reinstating the activity (Chevalley and Bangerter, 2010). Finally, it would be interesting to explore boundary conditions on participants’ tolerance for resource allocation to different tasks in joint multitasking. Depending on the communication medium, joint activities can be characterized by a greater or lesser degree of co-presence between participants, which may affect the means by which mutual orientation is displayed (Clark and Brennan, 1991). Moreover, the pace (Dix et al., 1998) of concurrent joint activities may vary (e.g., e-mail conversations have a slower pace than face-to-face conversations), thereby affecting the rate at which signals of mutual orientation to a conversation need to be deployed (and thus the duration for which they can be allocated to other, concurrent tasks).

Our findings expand our understanding of the complexity of resource allocation processes in multiple joint activities. More research is needed to better understand these processes, especially as multitasking often takes place in the context of joint activities. While current theories of multitasking (Salvucci and Taatgen, 2008) describe individual cognition in great detail, interpersonal coordination processes remain underspecified. Our findings suggest that particular resources like gaze can be allocated to both cognitive control processes as well as interpersonal coordination. Some of the constraints that govern resource allocation extend beyond the cognitive realm (e.g., gaze allocation is also sensitive to affiliational imperatives like demonstrating orientation to the conversation; Enfield, 2006), and theories of multitasking may have to be extended to account for these constraints.

## Author Contributions

EM participated in the study design, data collection, coding and analysis and preparation of the manuscript. AB participated in the study design, coding, analysis and preparation of the manuscript. Both authors agree with the publication of this work in its current version.

## Conflict of Interest Statement

The authors declare that the research was conducted in the absence of any commercial or financial relationships that could be construed as a potential conflict of interest.

## References

[B1] Akhtar N. Gernsbacher M. A. (2008). On privileging the role of gaze in infant social cognition. *Child Dev. Perspect.* 2 60–66. 10.1111/j.1750-8606.2008.00044.x PMC4266544 25520748

[B2] Argyle M. (1975). *Bodily Communication.* London: Methuen.

[B3] Argyle M. Dean J. (1975). Eye-contact, distance and affiliation. *Sociometry* 6 289–304. 14341239

[B4] Bangerter A. Chevalley E. Derouwaux S. (2010). Managing third-party interruptions in conversations: effects of duration and conversational role. *J. Lang. Soc. Psychol.* 29 235–244. 10.1177/0261927X09359591

[B5] Bangerter A. Clark H. H. (2003). Navigating joint projects with dialogue. *Cogn. Sci.* 27 195–225. 10.1207/s15516709cog2702_3

[B6] Bavelas J. B. Coates L. Johnson T. (2000). Listeners as co-narrators. *J. Pers. Soc. Psychol.* 79 941–952. 10.1037/0022-3514.79.6.941 11138763

[B7] Bavelas J. B. Coates L. Johnson T. (2002). Listener responses as a collaborative process: the role of gaze. *J. Commun.* 52 566–580. 10.1111/j.1460-2466.2002.tb02562.x

[B8] Brennan S. E. (2005). “How conversation is shaped by visual and spoken evidence,” in *Approaches to Studying World-Situated Language Use: Bridging the Language-as-Product and Language-Action Traditions* eds Trueswell J. Tanenhaus M. (Cambridge, MA: MIT Press) 95–129.

[B9] Brown P. Levinson S. (1987). *Politeness: Some Universals in Language Use.* Cambridge: Cambridge University Press.

[B10] Chevalley E. Bangerter A. (2010). Suspending and reinstating joint activities with dialogue. *Discourse Process.* 47 263–291. 10.1080/01638530902959935

[B11] Clark H. H. (1996). *Using Language.* Cambridge: Cambridge University Press.

[B12] Clark H. H. Bangerter A. (2004). “Changing conceptions of reference,” in *Experimental Pragmatics* eds Noveck I. Sperber D. (Basingstoke: Palgrave Macmillan) 25–49.

[B13] Clark H. H. Brennan S. A. (1991). “Grounding in communication,” in *Perspectives on Socially Shared Cognition* eds Resnick L. B. Levine J. M. Teasley S. D. (Washington, DC: APA Books).

[B14] Clark H. H. Fox Tree J. E. (2002). Using uh and um in spontaneous speaking. *Cognition* 84 73–111. 10.1016/S0010-0277(02)00017-3 12062148

[B15] Clark H. H. Krych M. A. (2004). Speaking while monitoring addressees for understanding. *J. Mem. Lang.* 50 62–81. 10.1016/j.jml.2003.08.004

[B16] Clark H. H. Schaefer E. F. (1989). Contributing to discourse. *Cogn. Sci.* 13 259–294. 10.1207/s15516709cog1302_7

[B17] Cook M. (1977). Gaze and mutual gaze in social encounters: how long—and when—we look others “in the eye” is one of the main signals in nonverbal communication. *Am. Sci.* 65 328–333.

[B18] Dix A. Ramduny D. Wilkinson J. (1998). Interaction in the large. *Interact. Comput.* 11 9–32. 10.1016/S0953-5438(98)00031-9

[B19] Enfield N. J. (2006). “Social consequences of common ground,” in *Roots of Human Sociality: Culture, Cognition and Interaction* eds Enfield N. J. Levinson S. C. (Oxford: Berg) 399–430.

[B20] Goodwin C. (1981). *Conversational Organization: Interaction between Speakers and Hearers.* New York, NY: Academic Press.

[B21] Goodwin C. Heritage J. (1990). Conversation analysis. *Annu. Rev. Anthropol.* 19 283–307. 10.1146/annurev.an.19.100190.001435

[B22] Haddington P. Keisanen T. Mondada L. Nevile M. (eds) (2014). *Multiactivity in Social Interaction: Beyond Multitasking.* Amsterdam: John Benjamins.

[B23] Hanna J. E. Brennan S. E. (2007). Speakers’ eye gaze disambiguates referring expressions early during face-to-face conversation. *J. Mem. Lang.* 57 596–615. 10.1016/j.jml.2007.01.008

[B24] Ho S. Foulsham T. Kingstone A. (2015). Speaking and listening with the eyes: gaze signaling during dyadic interactions. *PLoS ONE* 10:e0136905 10.1371/journal.pone.0136905 PMC4550266 26309216

[B25] Kendon A. (1967). Some functions of gaze-direction in social interaction. *Acta Psychol.* 26 22–63. 10.1016/0001-6918(67)90005-4 6043092

[B26] Kendon A. (1990). *Conducting Interaction: Patterns of Behavior in Focused Encounters.* Cambridge: Cambridge University Press.

[B27] Louwerse M. M. Dale R. A. Bard E. G. Jeuniaux P. (2012). Behavior matching in multimodal communication is synchronized. *Cogn. Sci.* 36 1404–1426. 10.1111/j.1551-6709.2012.01269.x 22984793

[B28] Macdonald R. G. Tatler B. W. (2013). Do as eye say: Gaze-cueing and language in a real-world social interaction. *J. Vis.* 13:6 10.1167/13.4.6 23479476

[B29] Mayor E. Bangerter A. (2013). “Coordinating turning while walking and talking,” in *Cooperative Minds: Social Interaction and Group Dynamics. Proceedings of the 35th Annual Meeting of the Cognitive Science Society* eds Knauff M. Pauen M. Sebanz N. Wachsmuth I. (Austin: Cognitive Science Society) 3002–3007.

[B30] Mayor E. Bangerter A. (2015). Managing perturbations during handover meetings: a joint activity framework. *Nurs. Open* 2 130–140. 10.1002/nop2.29 27708808 PMC5047324

[B31] McFarlane D. C. Latorella K. A. (2002). The scope and importance of human interruption in human–computer interaction design. *Hum. Comput. Interact.* 17 1–61. 10.1207/S15327051HCI1701_1

[B32] Mondada L. (2012). Talking and driving: multiactivity in the car. *Semiotica* 191 223–256. 10.1515/sem-2012-0062

[B33] Mondada L. (2014). Bodies in action: multimodal analysis of walking and talking. *Lang. Dialogue* 4 357–403. 10.1075/ld.4.3.02mon

[B34] Monsell S. (2003). Task switching. *Trends Cogn. Sci.* 7 134–140. 10.1016/S1364-6613(03)00028-7 12639695

[B35] Pennebaker J. W. Francis M. E. Booth R. J. (2001). *Linguistic Inquiry and Word Count (LIWC): A Computerized Text Analysis Program.* Mahwah, NJ: Erlbaum.

[B36] Richardson D. C. Dale R. Kirkham N. Z. (2007). The art of conversation is coordination: common ground and the coupling of eye movements during dialogue. *Psychol. Sci.* 18 407–413. 10.1111/j.1467-9280.2007.01914.x 17576280

[B37] Rossano F. (2012a). “Gaze in conversation,” in *The Handbook of Conversation Analysis* eds Sidnell J. Stivers T. (Malden, MA: Wiley-Blackwell) 308–329.

[B38] Rossano F. (2012b). *Gaze Behavior in Face-to-Face Interaction.* Nijmegen: Max Planck Institute for Psycholinguistics Series.

[B39] Sacks H. Schegloff E. A. Jefferson G. (1974). A simplest systematics for the organization of turn-taking for conversation. *Language* 50 696–735. 10.2307/412243

[B40] Salvucci D. D. Taatgen N. A. (2008). Threaded cognition: an integrated theory of concurrent multitasking. *Psychol. Rev.* 115 101–130. 10.1037/0033-295X.115.1.101 18211187

[B41] Shockley K. Richardson D. C. Dale R. (2009). Conversation and coordinative structures. *Topics Cogn. Sci.* 1 305–319. 10.1111/j.1756-8765.2009.01021.x 25164935

[B42] Sidnell J. Stivers T. (eds) (2005). Multimodal interaction [Special issue]. *Semiotica* 156 1–20.

[B43] Wu D. W.-L. Bischof W. F. Kingstone A. (2013). Looking while eating: the importance of the consequences of social interaction in social attention. *Sci. Rep.* 3:2356 10.1038/srep02356 PMC3733052 23912766

[B44] Zivotofsky A. Z. Hausdorff J. M. (2007). The sensory feedback mechanisms enabling couples to walk synchronously: an initial investigation. *J. NeuroEng. Rehabil.* 4 28 10.1186/1743-0003-4-28 PMC1973071 17686150

